# Addressing Noise and Skewness in Interpretable Health-Condition Assessment by Learning Model Confidence †

**DOI:** 10.3390/s20247307

**Published:** 2020-12-19

**Authors:** Yuxi Zhou, Shenda Hong, Junyuan Shang, Meng Wu, Qingyun Wang, Hongyan Li, Junqing Xie

**Affiliations:** 1 School of Electronics Engineering and Computer Science, Peking University, Beijing 100871, China; joy_yuxi@pku.edu.cn (Y.Z.); sjy1203@pku.edu.cn (J.S.); 1501111363@pku.edu.cn (M.W.); wangqingyun@pku.edu.cn (Q.W.); 2 Key Laboratory of Machine Perception, Ministry of Education, Peking University, Beijing 100871, China; 3 National Institute of Health Data Science at Peking University, Beijing 100191, China; hongshenda@pku.edu.cn; 4 Institute of Medical Technology, Health Science Center of Peking University, Beijing 100191, China; 5 Centre for Statistics in Medicine (CSM), Nuffield Department of Orthopaedics, Rheumatology and Musculoskeletal Sciences (NDROMS), University of Oxford, Oxford OX3 7LD, UK; junqing.xie@ndorms.ox.ac.uk

**Keywords:** noise, class skewness, model interpretability, deep learning, health-condition assessment

## Abstract

Assessing the health condition has a wide range of applications in healthcare, military, aerospace, and industrial fields. Nevertheless, traditional feature-engineered techniques involve manual feature extraction, which are too cumbersome to adapt to the changes caused by the development of sensor network technology. Recently, deep-learning-based methods have achieved initial success in health-condition assessment research, but insufficient considerations for problems such as class skewness, noisy segments, and result interpretability make it difficult to apply them to real-world applications. In this paper, we propose a K-margin-based Interpretable Learning approach for health-condition assessment. In detail, a skewness-aware RCR-Net model is employed to handle problems of class skewness. Furthermore, we present a diagnosis model based on K-margin to automatically handle noisy segments by naturally exploiting expected consistency among the segments associated with each record. Additionally, a knowledge-directed interpretation method is presented to learn domain knowledge-level features automatically without the help of human experts which can be used as an interpretable decision-making basis. Finally, through experimental validation in the field of both medical and aerospace, the proposed method has a better generality and high efficiency with 0.7974 and 0.8005 $F_{1}$ scores, which outperform all state-of-the-art deep learning methods for health-condition assessment task by 3.30% and 2.99%, respectively.

## 1. Introduction

Assessing the health condition of complex systems has been an exciting research area for a long time. It has a wide range of applications in healthcare, military, aerospace, and industrial fields. For instance, in the field of aerospace, the structural safety state of an aircraft could be assessed by detecting whether there is any structural damage, and thus the maintenance of the aircraft could be arranged reasonably before a plane crash happened because of the structural damage which is invisible to the human eye.

Based on the time-series data recorded by various sensors in the course of the operation of complex systems, machine learning methods can be applied to learn effective characteristics which are associated with health-condition assessment; thereafter, they can be used for detecting and assessing the health condition of the complex systems.

Health-condition assessors can be divided into two categories including feature-engineered methods and deep-learning-based methods. Before the process of feature-engineered methods, a pre-processing phase is required to get the domain handcraft features from the health-condition monitoring data. Although extracting effective features is difficult and time-consuming, many excellent works have proposed various features and achieved good performance [1,2,3,4,5,6,7,8]. For instance, Bornn et al. [9] used a support vector machine (SVM) combined with an autoregressive (AR) model for structural damage detection, and used signal reconstruction and residual estimation to locate the unhealthy ones. Carrara et al. [10] use extracted features to classify the cardiac disease by logistic regression, k-Nearest Neighbors, and random forests. A Bayesian-based approach is employed for health-condition assessment of bridge expansion joints [11].

Nevertheless, these kinds of methods highly rely on the domain expertise of users, as they have to manually extract features, which are the key to making the model distinguish between different health conditions, from the dataset before using the model. Consequently, it is difficult to propose a general framework for the health-condition assessment using traditional feature-engineered methods. Moreover, with the development of sensor network technology, time-series records generated by various sensors of complex systems are bursting in data volume, expanding in data dimensions, and decreasing in data value density. However, traditional feature-engineered techniques, which involve time-consuming manual feature extraction, are too cumbersome to adapt to such changes.

Recently, deep-learning-based methods like Convolutional Neural Network (CNN) [12,13,14,15,16], Recurrent Neural Network (RNN) [17,18,19,20], Convolutional Recurrent Neural Network (CRNN) [21,22,23], and Residual Neural Networks (Res-Net) [24,25] have achieved initial success in health-condition assessment research by extracting features automatically. For example, CNN is employed for the automatic classification of electroencephalographic signals to diagnose the disease of Alzheimer’s [26] and structural health monitoring of tall buildings subject to wind loads [27], while a CRNN-based approach is presented as a diagnostic tool for the classification of abnormal ECG signals [28].

Although the evaluation accuracy has been greatly improved compared with traditional methods [29,30,31], when facing problems in real-world applications, deep-learning-based health-condition assessment methods have insufficient considerations for problems such as class skewness, noisy segments, and result interpretability. These problems make it difficult to apply their success to real-world applications.

**Figure 1:**
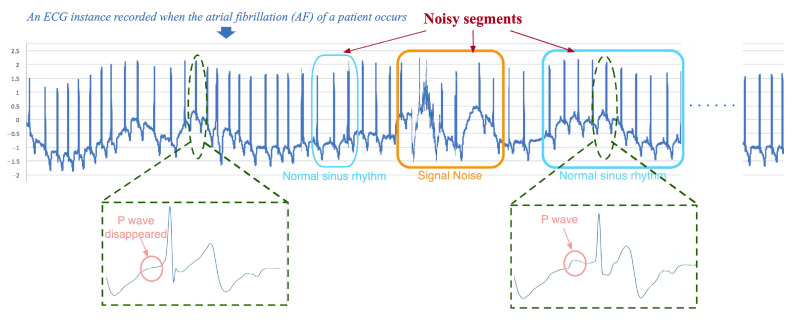
An example of a health-condition monitoring record with noisy segments whose label is Atrial Fibrillation.

- **Class skewness:** As unhealthy conditions (e.g., diseases) occur rarely, the existence of class imbalance in health-condition monitoring records is inevitable. Classifying imbalanced data is a challenging problem [32]. Directly applying a machine learning algorithm may result in poor performance on the minority classes, as they are prone to ignore the subtle influence on the accuracy of an algorithm [33].
- **Noisy segments:** Most methods employ noise elimination approaches (e.g., grey spectral noise estimation [34], Dense Neural Network + spectrum-based noise elimination [35], CNN + Kalman filter [27], Encoder–Decoder [36], etc.) to denoise health-condition monitoring data, but they cannot deal with all of the noisy segments effectively, as the noisy segments refer to not only signal noise, but also the segments containing noisy labels. Taking a real-world electrocardiograph (ECG) record which is shown in Figure 1 as an example, its reference health-condition label given by cardiologists is ‘Atrial Fibrillation’. However, it may still contain some other types of segments (e.g., Normal sinus rhythm, etc.). Different from the irregular changes of the amplitude of segments with signal noises in the frequency domain or non-periodic variation in the time domain, only very subtle key differences in the segments that contain noisy labels can be found. For example, as shown in Figure 1, the morphological features of segments containing the noisy label of ‘Normal sinus rhythm’ are very similar to that containing only the ground-truth label of ‘Atrial fibrillation’, except that a ‘P wave’ only appears in the waveform of segments that contain the noisy label of ‘Normal sinus rhythm’. However, the traditional noise elimination methods cannot effectively deal with these segments with noisy labels which are very similar to that containing only reference labels in morphological features. Furthermore, in the field of health-condition assessment, the accuracy of the model may be greatly reduced when a monitoring record contains too many noises, and thus it may lead to sudden death, air crash, and other tragic events due to the misdiagnosis. Therefore, in addition to accurate health-condition detection from the good-quality monitoring data, automatic recognition of low-quality noisy signals is required as well to remind domain experts to intervene in time. Nevertheless, the use of noise elimination methods may affect the accuracy of the detection of low-quality noisy signals, as it may cause changes or losses of features representing the quality of the monitoring signal itself.
- **Interpretability of prediction results:** Most existing deep-learning-based methods are often regarded as black-box models, as they can only output a specific diagnosis result without necessary interpretability. Nevertheless, in the real-life scenario of health-condition assessment, to realize “why” is, for the most part, more important. To explain the results of Deep Neural Network (DNN) models, most methods use the attention mechanism to highlight segments of an input record which are strongly associated with the model prediction and consider them as an explanation of prediction results [37,38], while others measure the role of each feature in the prediction process [39,40]. However, these kinds of highlighted segments and exploration of feature effects can only provide auxiliary information, which cannot be a decision-making basis actually, as they cannot provide domain technological proofs. For instance, to achieve a sound balance between the best commercial and safety decision-making carefully, airlines may require detailed technological information on why the flight plan of an aircraft has to be suspended immediately. Thus, interpretable explanations of the model must be technological proofs with admissive knowledge which can be widely accepted by domain experts.

Accordingly, there is a definite need for accurate, general, and interpretable health-condition monitoring solutions that can automatically deal with class skewness and noisy segments without changing or losing features representing the quality of the monitoring signal itself. In this paper, we propose a K-margin-based intErpretable lEarNing (KEEN) for interpretable health-condition assessment.

- We first employ a skewness-aware RCR-Net model to establish a general deep learning model, which can automatically obtain the features and handle problems of class skewness in a wide range of health-condition monitoring data.
- A diagnosis model based on K-margin is presented to automatically focus on the most essential segments associated with a monitoring record, and tackle noisy segments by using the expected consistency among the segments which are related to the same record. The classification of each monitoring record can be identified by selecting the most likely label of the relevant top-K augmented segments.
- A knowledge-directed interpretation method is proposed to learn how to extract features that can be regarded as important domain knowledge without the help of human experts from the time-series data automatically. As a result, one can use these domain knowledge-level features as an interpretable decision-making basis.
- Thorough experiments are carried out on Atrial Fibrillation Monitoring dataset [41] and Structural Damage Monitoring Dataset [42]. The experimental results demonstrate that the proposed method with 0.7974 and 0.8005 $F_{1}$ scores, respectively, which outperform all state-of-the-art deep learning methods for health-condition assessment tasks by 3.30% and 5.71%.

## 2. Methods

### 2.1. Problem Definition and General Framework

Health-condition assessment is the task of automatically classifying a health-condition monitoring data into one of health-condition classes. Formally, we denote the training dataset as D={X,Y,Z} where $X=\{x^{\left(1\right)},x^{\left(2\right)},\ldots ,x^{\left(N\right)}\}$ are labelled health-condition monitoring sequence inputs, $Y=\{y^{\left(1\right)},y^{\left(2\right)},\ldots ,y^{\left(N\right)}\}$ the corresponding label set, $Z=\{z^{\left(1\right)},z^{\left(2\right)},\ldots ,z^{\left(N\right)}\}$ the set of corresponding domain knowledge attribute set, each domain knowledge attribute set $z^{\left(i\right)}=\left\{z^{(i)1},z^{(i)2},\ldots ,z^{(i)F}\right\}$ has *F* domain knowledge attributes and *N* the total number of training data. Moreover, each health-condition monitoring record $x^{\left(i\right)}$ has *L* monitoring channels $x=\{x^{(i)1},x^{(i)2},\ldots ,x^{(i)L}\}$ and each channel is a time-series $x^{(i)l}=\left\{x_{1}^{(i)l},x_{2}^{(i)l},\ldots \right\}$.

Given the unlabelled testing dataset $D_{⊓}=\left\{X\right\}$ where $X=\{x^{\left(1\right)},x^{\left(2\right)},\ldots ,x^{\left(N\right)}\}$ are unlabelled health-condition monitoring sequence inputs, the goal of the health-condition assessment is to learn a predictive model which takes unlabelled health-condition monitoring sequence $x^{\left(i\right)}$ as input and outputs the prediction of its domain knowledge attributes ${\hat{z}}^{\left(i\right)}$ and its prediction of health-condition class ${\hat{y}}^{\left(i\right)}\in C$ where $C=\{c_{1},c_{2},\ldots ,c_{M}\}$ is a set of *M* different health-condition classes.

**Figure 2:**
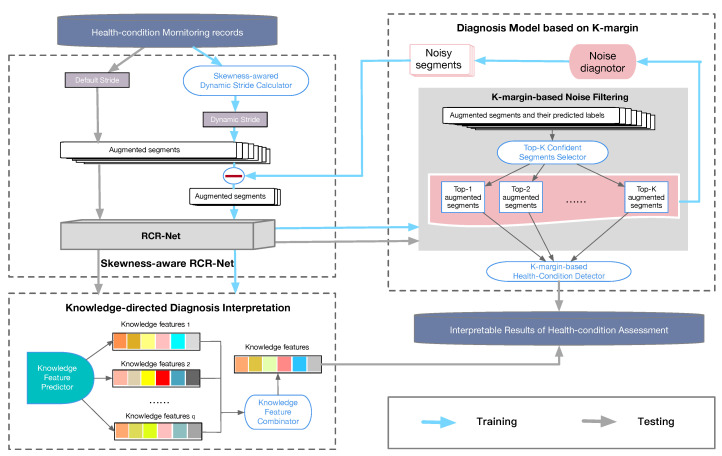
The framework of the k-margin-based interpretable model.

As research results based on deep learning have achieved remarkable success in health-condition assessment tasks recently, which can naturally integrate and extract hierarchical features, the DNN model is used as the basic classifier. Moreover, to automatically extract features from CNN and capture long-term trends from RNN, we use a residual convolution recurrent neural network (RCR-Net), as shown in Figure 2.

Nevertheless, a RCR-Net model still cannot be directly applied for reliably detecting and assessing health conditions from monitoring data with noise and class imbalance. On the one hand, the class imbalance and semantical ambiguities caused by noisy segments may lead to poor and even unacceptable quality of DNN models. On the other hand, a proper explanation of the health-condition assessment result is required to support reliable decision-making.

A K-margin-based interpretable learning method is presented to solve the above difficulties. Specifically, a skewness-aware RCR-Net approach (Section 2.2) is presented to alleviate the problem of class imbalance. In addition, to tackle noise, a K-margin based diagnosis model (Section 2.3) is proposed, which can automatically focus on the most important segments and involve only part of the labeled segments in the skewness-aware RCR-Net learning process. Finally, a knowledge-directed diagnosis interpretation model is employed to extract features that can be regarded as important domain knowledge. The framework of our model is shown in Figure 2.

### 2.2. Skewness-Aware RCR-Net

To alleviate the problems of the lack of well-labelled data and class imbalance, a skewness-aware RCR-Net model is proposed to deal with these problems. First, a skewness-aware data augmentation is employed to generate more short-term segments from the long-term monitoring records. After that, a multi-view RCR-Net model is presented to establish a general deep learning model for capturing features automatically from the health-condition monitoring data.

Generally, two predefined parameters are required for the skewness-aware data augmentation—the length of the sliding windows W) and the maximum stride threshold M. For records with rare labels, the dynamic stride S becomes smaller, while, for records with common labels, it becomes larger. Formally, given the maximum stride threshold of M and the label set $C=\{c_{1},c_{2},\ldots ,c_{M}\}$, the dynamic stride of records with labeled $C_{j}$ is given by the following formula:

(1) $$\begin{array}{c} S_{c_{j}}=\left\lceil M\times \frac{\;|\mathrm{records}\;\mathrm{labelled}\;c_{j}\;|}{\max_{s=1}^{M}\;\left|\mathrm{records}\;\mathrm{labelled}\;c_{s}\;\right|}\right\rceil \end{array}$$

Please note that, if the length of a monitoring record is less than the length of the sliding windows W, a zero-padding approach would be employed to pad it with zeros at the end of it

Additionally, a multi-view RCR-Net is presented to establish a general deep learning model, which can automatically obtain features for a wide range of health conditions. Specifically, it consists of 33 layers of residual blocks [43], one layer of recurrent block, and one layer of a fully connected block. The purpose of using residual blocks is to construct a deeper model through the residual connection between blocks, and automatically extract more effective local morphological-level features. To capture potential trend-level features in monitoring data, a recurrent layer with Bi-directional Long-Short Term Memory (Bi-LSTM) cells is employed. Finally, the prediction is made by a fully connected layer and a softmax layer. The cross-entropy loss of the objective function is calculated to optimize the loss of the training neural network.

**Figure 3:**
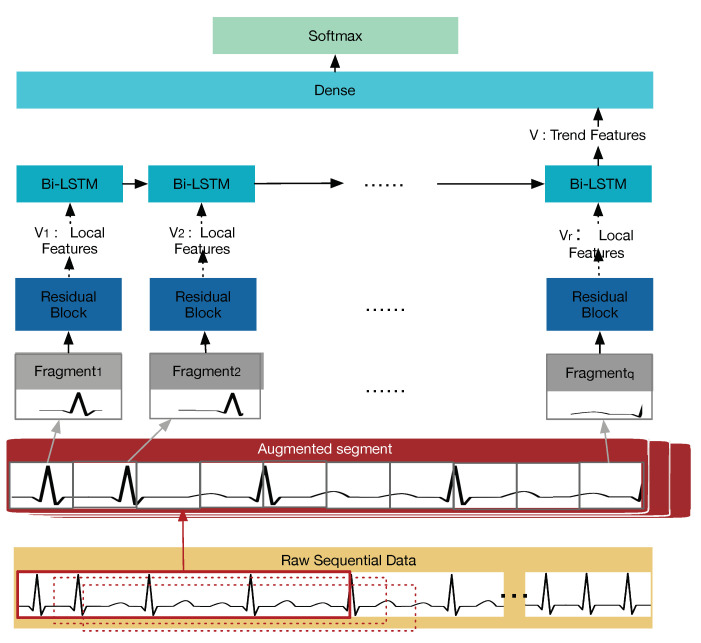
The high-level architecture of the multi-view RCR-Net model.

The high-level architecture of the multi-view RCR-Net is shown in Figure 3. It takes an augmented segment as input. After that, it splits the augmented segments into $\left\lceil \frac{W}{N}\right\rceil$ fragments with length of N and outputs the health-condition prediction of the augmented segment.

### 2.3. Diagnosis Model Based on K-Margin

**Figure 4:**
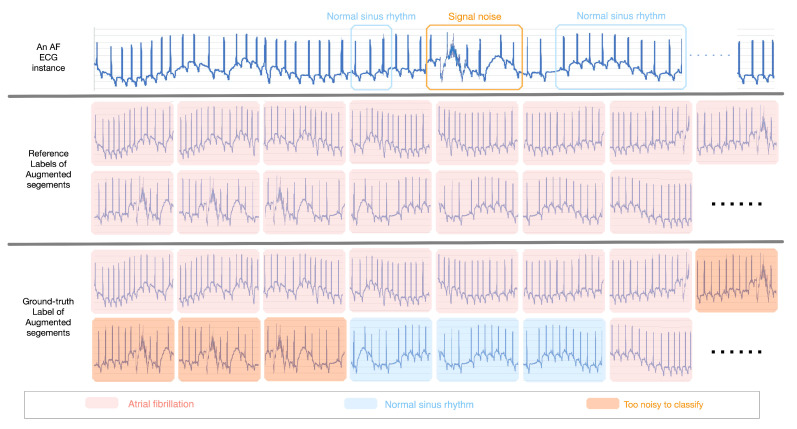
An example of “hard” segments with noisy labels caused by the data augmentation process.

Although the improvement of data inadequacy and class imbalance through data augmentation process is essential to enhancing the performance of the health-condition assessment model, it inevitably generates “hard” health-condition monitoring segments due to noisy segments. For example, as shown in Figure 4, the reference label of the 8th–14th augmented segments is ‘Atrial Fibrillation’ as the reference label of the ECG instance is ‘Atrial Fibrillation’. Nevertheless, the main ECG signs are ‘Too noisy to classify’ for the 8th–11th augmented segments and ‘Normal sinus rhythm’ for the 12th–14th augmented segments. Therefore, to filter these noisy segments, we first enhance the robustness of our model by using a K-margin-based noise filtering approach to compute the cross-entropy objective function of only a portion of the selected segments. Moreover, a K-margin-based health-condition Detector is proposed to predict the health condition of a monitoring record according to the top-K confident segments.

#### 2.3.1. K-Margin-Based Noise Filtering

A minimum uncertainty margin is proposed to select appropriate segments to calculate cross-entropy. As the prediction of all augmented segments of each health monitoring record can be obtained by a trained multi-view RCR-Net model, and thus we define the uncertainty margin of *t*-th segment $x_{t}^{\left(i\right)}$ for a given monitoring record $x^{\left(i\right)}$ as:

(2) $$\begin{array}{c} Margin\left(x_{t}^{\left(i\right)}\right)=P\left({\ddot{\hat{y}}}_{t}^{\left(i\right)}|x_{t}^{\left(i\right)}\right)\;-\;P\left({\dot{\hat{y}}}_{t}^{\left(i\right)}|x_{t}^{\left(i\right)}\right) \end{array}$$

where ${\dot{\hat{y}}}_{t}^{\left(i\right)}$ and ${\ddot{\hat{y}}}_{t}^{\left(i\right)}$ are the most probable and second-most probable prediction classes of the record $x_{t}^{\left(i\right)}$.

Usually, the trained model has less doubt (that is, more confidence) in distinguishing the two most probable categories if the uncertainty margin is smaller for a given health monitoring record $x_{t}^{\left(i\right)}$. Instead, segments with larger uncertainty margins are more ambiguous. Therefore, the most confident segment of *i*-th monitoring record $x^{\left(i\right)}$ can be defined as:

(3) $$\begin{array}{c} x_{*}^{\left(i\right)}=\underset{x_{t}^{\left(i\right)},t\in \left\{1,2,\ldots ,T\right\}}{\arg\min}\left(Margin\left(x_{t}^{\left(i\right)}\right)\right) \end{array}$$

where $x_{t}^{\left(i\right)}$ is the *t*-th segment of the record $x_{i}$. A diagnosis based on minimum uncertainty margin is a strategy to find the predicted class of a monitoring record with the largest confidence. Similarly, the most confident label of a record $x^{\left(i\right)}$ can be defined as follows:

(4) $$\begin{array}{c} {\hat{y}}_{*}^{\left(i\right)}=\underset{{\dot{\hat{y}}}_{t}^{\left(i\right)},t\in \left\{1,2,\ldots ,T\right\}}{\arg\min}\left(P\left({\ddot{\hat{y}}}_{t}^{\left(i\right)}|x_{t}^{\left(i\right)}\right)-P\left({\dot{\hat{y}}}_{t}^{\left(i\right)}|x_{t}^{\left(i\right)}\right)\right) \end{array}$$

A K-margin-based health-condition label prediction algorithm (K-margin) is presented (see Algorithm 1) to select top-K most confident segments for training the multi-view RCR-Net. Given a health-condition monitoring record $x^{\left(i\right)}$ and its augmented segments $x^{\left(i\right)}=\left\{x_{1}^{\left(i\right)},x_{2}^{\left(i\right)},\ldots ,x_{T}^{\left(i\right)}\right\}$, it requires *K* iterations to output the top-K most confident segments $X_{1:K}^{\left(i\right)}$ and their label ${\hat{Y}}_{1:K}^{\left(i\right)}$ under trained multi-view RCR-Net model ϕ.

Intuitively, the top-K most confident fragments $X_{1:K}^{\left(i\right)}$ can be used for the multi-view RCR-Net training process, instead of the entire segments array $x^{\left(i\right)}$, to avoid learning features from noise segments and automatically focus on the most essential part that can represent the characteristics of a given record. Nevertheless, it is not reliable to use them for the training process when the model is not reliable yet, since they are selected by the KEEN model. Hence, we calculate the average probability of predictions for all augmented segments of a record $x^{\left(i\right)}$:

(5) $$\begin{array}{c} \alpha_{i}\;=\frac{1}{T}\;\;\left(\sum_{t=1}^{T}P\left({\dot{\hat{y}}}_{t}^{\left(i\right)}|x_{t}^{\left(i\right)}\right)\right) \end{array}$$

where $P({\dot{\hat{y}}}_{t}^{\left(i\right)}|x_{t}^{\left(i\right)})$ is the prediction probability of *t*-th augmented candidate for a record $x^{\left(i\right)}$, and *T* is the number of augmented candidates for the record $x^{\left(i\right)}$. The top-K most confident candidates for the given record $x_{1:K}^{\left(i\right)}$ would be used in the model fine-tuning process to achieve higher consistency among the augmented candidates and reduce the impact of noisy ones when $\alpha_{i}>5$. Otherwise, all the candidates would be used. The reason is that the low average probability of predictions for all augmented candidates indicates a poor model performance. Therefore, the multi-view RCR-Net model is not accurate and reliable, a more “hard” sample is required for the training process. Thereafter, the selected alpha-segments $X_{\alpha }^{\left(i\right)}$ can be defined as follows:

(6) $$\begin{array}{c} X_{\alpha }^{\left(i\right)}\;=\left\{\begin{array}{cc} X_{1:K}^{\left(i\right)}, & \mathrm{if}\;\alpha_{i}>0.5\\ x^{\left(i\right)}, & \mathrm{otherwise} \end{array}\right. \end{array}$$

Consequently, the task of health-condition assessment using our KEEN model can be expressed as optimizing the cross-entropy objective function:

(7) $$\begin{array}{c} L\left(X,\hat{y}\right)=\frac{1}{N\cdot |X_{\alpha }^{\left(i\right)}|}\sum_{i=1}^{N}\sum_{t=1}^{|X_{\alpha }^{\left(i\right)}|}\log P\left(Y={\hat{y}}_{t}^{\left(i\right)}|X=X_{\alpha t}^{\left(i\right)}\right) \end{array}$$

**Algorithm 1** K-margin($x^{\left(i\right)},K,\phi$)
**Input**: A *T*-segments array $x^{\left(i\right)}=\{x_{1}^{\left(i\right)},x_{2}^{\left(i\right)},\ldots ,x_{T}^{\left(i\right)}$} of an input health-condition monitoring record $x^{\left(i\right)}$, trained multi-view RCR-Net model ϕ**Parameter**: An integer K**Output**: Top-K most confident segments $X_{1:K}^{\left(i\right)}$ and their label predictions ${\hat{Y}}_{1:K}^{\left(i\right)}$
1:$x^{{}^{\prime }\left(i\right)}\leftarrow x^{\left(i\right)}$2:$X_{1:K}^{\left(i\right)}\leftarrow \emptyset$, ${\hat{Y}}_{1:K}^{\left(i\right)}\leftarrow \emptyset$, k←K3:**while**k>0**do**4: $x_{*}^{\left(i\right)}\leftarrow \mathrm{Most}\;\;\mathrm{confident}\;\;\mathrm{segment}\;\;\mathrm{predicting}\;\;\mathrm{by}\;\phi \left(x^{{}^{\prime }\left(i\right)}\right)$ using Equation (3)5: ${\hat{y}}_{*}^{\left(i\right)}\leftarrow \mathrm{Most}\;\;\mathrm{confident}\;\;\mathrm{label}\;\;\mathrm{predicting}\;\;\mathrm{by}\;\phi \left(x^{{}^{\prime }\left(i\right)}\right)$ using Equation (4)6: $x^{{}^{\prime }\left(i\right)}\leftarrow x^{{}^{\prime }\left(i\right)})\backslash x_{*}^{\left(i\right)}$ # Implementation: remove segment index of $x_{*}^{\left(i\right)}$ from segment indexes list of $x^{{}^{\prime }\left(i\right)}$7: $X_{1:K}^{\left(i\right)}\leftarrow X_{1:K}^{\left(i\right)}\cup \left\{x_{*}^{\left(i\right)}\right\}$, ${\hat{Y}}_{1:K}^{\left(i\right)}\leftarrow {\hat{Y}}_{1:K}^{\left(i\right)}\cup \left\{{\hat{y}}_{*}^{\left(i\right)}\right\}$8: k←k−19:**end while**10:**return**$X_{1:K}^{\left(i\right)},\;{\hat{Y}}_{1:K}^{\left(i\right)}$


#### 2.3.2. K-Margin-Based Health-Condition Detector

In order to diagnose the health-condition for the given health-condition monitoring record $x^{\left(i\right)}$, given the training model Φ, we convert the label predictions ${\hat{Y}}_{1:K}^{\left(i\right)}$ of the top-K most confident segments into the matrix ${\hat{Y}}_{1:K}^{\left(i\right)}\in R^{K\times M}$ as follows:

(8) $$\begin{array}{c} {\hat{Y}}_{k,j}^{\left(i\right)}=\left\{\begin{array}{cc} 1, & \mathrm{if}\;c_{j}=\underset{1\leq j\leq M}{\arg\max}\;{\hat{Y}}_{k,j}^{\left(i\right)}\\ 0, & \mathrm{otherwise} \end{array}\right. \end{array}$$

Furthermore, a K-majority weighted voting algorithm based on the minimum uncertainty margin is presented to output the most likely label of a given record $x^{\left(i\right)}$. The k-majority weighted voting method is to vote on the classes that the record $x^{\left(i\right)}$ may belong to, which can be defined as follows:

(9) $${\hat{y}}^{\left(i\right)}=\underset{c_{j}\in C}{\arg\max}\left(\sum_{1}^{K}{\hat{Y}}_{kj}^{\left(i\right)}\right)$$

Thus far, by naturally exploiting expected consistency among the segments associated with each record, our KEEN model can make a diagnosis of a given record $x^{\left(i\right)}$. It can automatically deal with noise as only a portion of segments that belong to the same record would be included in the learning process; thereafter, diagnosis would be made according to the predictions of the most essential segments as well.

### 2.4. Knowledge-Directed Interpretation

In the real-life scenario of health-condition assessment, the method of computer-aided diagnosis requires a high degree of interpretability so that humans can give a reliable conclusion based on the diagnosis basis.

Recently, some methods try to explain the DNN model by highlighting the most relevant segments of health-condition monitoring data [38] and exploring feature effects [40] in the prediction process. Nevertheless, this kind of method cannot provide detailed domain technological-level information on “why”, as we still do not know the relationship between this kind of explanation and domain knowledge.

The main goal of knowledge-directed interpretation is to provide an indispensable domain technological-level information on “why”, and to make the complex reliability of predicting results well explicable in order to make reliable decisions. Specifically, we integrate domain knowledge into the training process of the skewness-aware RCR-Net.

The function of knowledge-directed interpretation is similar to the observation process when domain experts try to classify health-condition monitoring data. When domain experts diagnose health-condition monitoring records, they first observe the characteristics of these records. Taking arrhythmia diagnosis as an example, cardiologists usually analyze the ECG record to see characteristics such as “P waves disappear”, “RR-interval”, etc. After that, using these characteristics to classify them. Inspired by this, a knowledge-directed interpretation method is proposed to convert the morphological-level local features, which are automatically extracted from the health-condition monitoring record, into the knowledge-level features.

**Figure 5:**
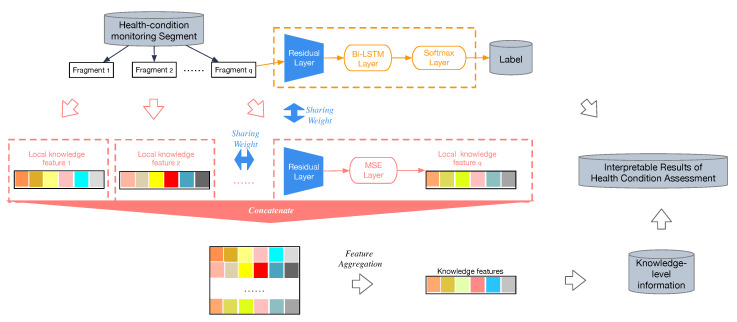
The architecture of a knowledge-directed interpretation method.

The architecture of the knowledge-directed interpretation method is shown in Figure 5. The health-condition predictor is shown in the orange dashed box, which trains a multi-view RCR-Net for health-condition classification. The red one is a knowledge-level feature extraction step, which shares weights with the Residual layer of health-condition predictor, and then replaces the Bi-LSTM layer and Softmax layer with a Mean square error loss (MSE) layer to form a knowledge-level feature extractor.

It takes a fragment $x_{tq}^{\left(i\right)}$ of a segment $x_{t}^{\left(i\right)}$ which is augmented from a health-condition monitoring recode $x^{\left(i\right)}$, and outputs the prediction of its knowledge-level features ${\hat{z}}_{tq}^{\left(i\right)}$. Subsequently, we concatenate all knowledge-level features of the fragments which belong to the same segment $x_{t}^{\left(i\right)}$ to get the combined features.

Formally, given extracted features ${\hat{z}}_{t}^{\left(i\right)}=\left\{{\hat{z}}_{t1}^{\left(i\right)},\ldots ,{\hat{z}}_{tq}^{\left(i\right)}\right\}$ corresponding to one segment $x_{t}^{\left(i\right)}$, where each ${\hat{z}}_{tq}^{\left(i\right)}\in R^{F}$, and ${\hat{z}}_{t}^{\left(i\right)}\in R^{q\times F}$. The aggregated features are computed by column-wise aggregation operation of ${\hat{z}}_{tq}^{\left(i\right)}$. Then, the *f*-th knowledge-level feature of a segment $x_{t}^{\left(i\right)}$ can be denoted as:

(10) $$\begin{array}{c} {\hat{z}}_{t}^{(i)f}=\psi_{f}\left(\left\{{\hat{z}}_{tq}^{\left(i\right)}\;|\;x_{tq}^{\left(i\right)}\in x_{t}^{\left(i\right)}\right\}\right) \end{array}$$

where $\psi_{f}\left(\cdot \right)$ is an aggregation operation. Usually, pooling operation (e.g., sum, max, average, etc.) [44] can be used to aggregate these features. As each of the knowledge-level features varies very much in nature, it requires aggregating them into one unified feature vector according to their own properties. To name only a few, a Max Pooling operation would be employed as the aggregation operation for the knowledge-level attribute of “P waves disappear” to determine whether there is “P wave disappear” in some fragments of a segment. On the contrary, Mean Pooling operation would be better for the knowledge-level attribute of “ST slope”. Sometimes, other aggregation operations may be used as well; for example, the calculation of the standard deviation coefficient may be employed for the knowledge-level attribute of “PR interval” to determine whether the interval is unequal on the entire record.

Similarly, the f-th knowledge-level feature of a record $x^{\left(i\right)}$ can be denoted as:

(11) $$\begin{array}{c} {\hat{z}}^{(i)f}=\psi_{f}\left(\left\{{\hat{z}}_{t}^{\left(i\right)}\;|\;x_{t}^{\left(i\right)}\in x^{\left(i\right)}\right\}\right) \end{array}$$

To avoid learning knowledge-level information from noisy segments, the aggregation operation would be only applied on the selected top-K most confident segments for the given health-condition monitoring record $x^{\left(i\right)}$ under trained model ϕ. We chose the mean square error loss as the empirical loss; thereafter, we optimize the knowledge-directed interpretation model by gradient descent as follows:

(12) $$\begin{array}{c} L\left(X,\hat{z}\right)=\frac{1}{N\cdot |X_{\alpha }^{\left(i\right)}|\cdot F}\sum_{i=1}^{N}\sum_{t=1}^{|X_{\alpha }^{\left(i\right)}|}\sum_{f=1}^{F}{\left(z_{t}^{(i)f}-{\hat{z}}_{t}^{(i)f}\right)}^{2} \end{array}$$

The total loss could be defined as follows:

(13) $$\begin{array}{c} L\left(X,\hat{y},\hat{z}\right)=\frac{1}{N\cdot |X_{\alpha }^{\left(i\right)}|}\sum_{i=1}^{N}\sum_{t=1}^{|X_{\alpha }^{\left(i\right)}|}\log P\left(Y={\hat{y}}_{t}^{\left(i\right)}|X=X_{\alpha t}^{\left(i\right)}\right)+\frac{1}{N\cdot |X_{\alpha }^{\left(i\right)}|\cdot F}\sum_{i=1}^{N}\sum_{t=1}^{|X_{\alpha }^{\left(i\right)}|}\sum_{f=1}^{F}{\left(z_{t}^{(i)f}-{\hat{z}}_{t}^{(i)f}\right)}^{2} \end{array}$$

In this way, domain technological-level features (e.g., P waves disappear, RR-interval, etc.), which correspond to domain knowledge that is convincing and understandable to a domain expert, can be extracted.

## 3. Experiments

### 3.1. Dataset

We carry out experiments on two datasets:

- Atrial Fibrillation Monitoring Dataset [41]: 8528 ECG monitoring records, which last from 9 s to slightly more than 60 s, is contained. All ECG records were sampled as 300 Hz and they have been band pass filtered by the AliveCor device. However, there are still many signal noises contained in this dataset. To filter the low-quality monitoring signals, all monitoring records which contain too many signal noises would be classified into a category called “Too noisy to classify”. Moreover, all health conditions that are abnormal but not an atrial fibrillation (e.g., junctional arrhythmia, ventricular arrhythmia, etc.) are considered as a single category, that is, other rhythms. As a result, these records are divided into four categories of health conditions: (1) Normal sinus rhythm **N** (contains 5050 records, accounting for about 59.22%), (2) Atrial Fibrillation **A** (contains 738 records, accounting for about 8.65%), (3) Other rhythm **O** (contains 2456 records, accounting for about 28.80%), and (4) Too noisy to classify **P** (contains 284 records, accounting for about 3.33%).
- Aircraft Monitoring Dataset [42]: The aircraft structural damage monitoring dataset contains 808 labeled structural damage testing records lasting about 1 s sampling with 10 MHz. Each record has two channels—the baseline channel and monitoring channel. The data of the baseline channel were collected before the four-point bending test for composite laminates and impact test for stiffened plate were carried out. In addition, the data of the monitoring channel were collected during the four-point bending test and impact test. These records are classified as 2 health-condition class: (1) Normal **N** (contains 538 records, accounting for about 66.58%), (2) Structural damage **D** (contains 270 records, accounting for about 33.42%).

### 3.2. Performance Measurements

Several common metrics (Precision and Recall) are used to measure the model performance by evaluating the proximity of the predicted labels to the referenced labels given by domain experts:

- $Precision\;=\frac{1}{M}\sum_{c=1}^{M}\sum_{i\in \{i|y^{\left(i\right)}=c\}}\frac{𝟙(y^{\left(i\right)}={\hat{y}}^{\left(i\right)})}{\left|\{i|y^{\left(i\right)}=c\}\right|}$.
- $Recall\;=\frac{1}{M}\sum_{c=1}^{M}\sum_{i\in \{i|y^{\left(i\right)}=c\}}\frac{𝟙(y^{\left(i\right)}={\hat{y}}^{\left(i\right)})}{\left|\{i|{\hat{y}}^{\left(i\right)}=c\}\right|}$.

In addition, the following $F_{1}$ evaluation measurements are employed for the Atrial Fibrillation Monitoring dataset:

- *$F_{1}$ scores of each class*: Denoted as $F_{1N}$ for normal sinus rhythm, $F_{1A}$ for AF, $F_{1O}$ for other rhythm, $F_{1P}$ for noise. Detailed definitions can be found in [41].
- *Averages of $F_{1}$ scores*: $F_{1}=\frac{F_{1N}+F_{1A}+F_{1O}+F_{1P}}{4}$.

Similarly, the following $F_{1}$ evaluation measurements are employed for the Aircraft Monitoring Dataset by using the counting rules shown in Table 1:

- *$F_{1}$ scores of Normal class*: $F_{1N}=\frac{2\cdot N_{n}}{\sum_{N}+\sum_{n}}$
- *$F_{1}$ scores of Structural Damage class*: $F_{1D}=\frac{2\cdot D_{d}}{\sum_{D}+\sum_{d}}$
- *Averages of $F_{1}$ scores*: $F_{1}=\frac{F_{1N}+F_{1D}}{2}$

### 3.3. Implementation Details

The above performance measurements are used to evaluate the effectiveness of various state-of-the-art DNN methods and reported average results by running a 5-fold cross validation. Furthermore, the knowledge-level attributes (P wave disappear, P wave amplitude, QRS complex amplitude, QRS duration, T wave amplitude, and PR interval, and ST slope) are extracted from the Atrial Fibrillation Monitoring dataset using the ECGPUWAVE [45] on each fragment and the knowledge-level attributes of each segment and record are generated by aggregation operations to train the knowledge-directed interpretation model. Similarly, the knowledge-level attributes (Peak-to-peak amplitude [46], Pearson correlation coefficient [47], and Signal energy [48]) are extracted from the Aircraft Monitoring Dataset.

For the training dataset, the parameter value of W and max stride threshold M is set to be 6000 and 500 for skewness-aware data augmentation, respectively—while, for the testing dataset, as their labels are unknown, a sliding window with a window size W=6000 would still be used for generating the segments, but its stride would be set to a default value of 300. In addition, the parameter values of *K* and N are set to be 3 and 300, respectively, for training the KEEN (without knowledge-directed interpretation) model and KEEN+ (including knowledge-directed interpretation) model (See Section 2.3) which are implemented on Tensorflow r1.4 using Python 3.6.2.

### 3.4. Comparing with Other Methods

The following state-of-the-art deep neural network methods (their shape of input and output are similar to the Atrial Fibrillation Monitoring dataset and Aircraft Monitoring Dataset to avoid the changes of model architecture) are compared with the proposed methods:

- *CNN* uses a variation of multilayer perceptrons to automatically extract features. Recently, Sodmann et al. [49] constructed CNN architecture for health-condition assessment without manually extracted features.
- *RNN* can automatically extract the time-domain trend features by allowing it to exhibit temporal dynamic behavior. It is used as the major classifier for health-condition assessment in Faust et al. [20].
- *CRNN* takes advantage of convolutional neural networks (CNNs) for local feature extraction and recurrent neural networks (RNNs) for temporal summarization of the extracted features. The CRNN model is employed in Liu et al. [22] for detecting the unhealthy condition of myocardial infarction (MI).
- *ResNet* can build very deep networks through modules called residual layers, which can avoid higher errors rate due to naively adding CNN layers [43]. In Hannun et al. [24], it is employed for cardiac health-condition assessment, and the accuracy is comparable to or higher than that of cardiologists.

## 4. Results Discussion

### 4.1. Effect of Application in the Field of Medical Health

To validate the accuracy of the proposed methods in the medical field, the Atrial Fibrillation Monitoring dataset, which contains ECG records sampled from 8528 selected patients in real-world scenarios, is used for evaluating the effectiveness of our methods for the task of atrial fibrillation diagnosis.

#### 4.1.1. Effectiveness of Atrial Fibrillation Diagnosis

**Figure 6:**
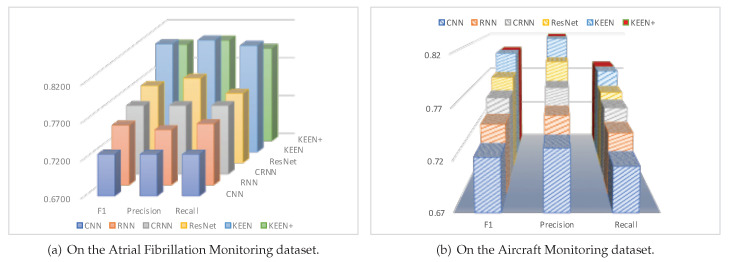
The result of Precision, Recall, F1 scores.

It is clear from the diagrams in Figure 6a that the precision, recall, and $F_{1}$ scores of the KEEN and KEEN+ are better than all the state-of-the-art deep learning methods in the task of atrial fibrillation diagnosis. The methods of CNN, RNN, RCNN, and RESNET are not effective, mainly due to the high noise contamination in this data set, and such methods do not deal with the problem of noisy segments. The method of ResNet works better mainly because of deeper networks. However, it is limited by the ability of model expression of the first three methods to build very deep networks, which may lead to the information loss and feature loss.

Additionally, the $F_{1}$ scores on the Atrial Fibrillation Monitoring dataset are demonstrated in Table 2. We can see that it is an effective method to improve prediction performance by automatically focusing on the most essential segments, as the $F_{1}$ scores of KEEN and KEEN+ are 0.8125 and 0.7974 respectively, which are 5.26–12.08% and 3.30–10.00% higher than other DNN methods. Remarkably, compared to other DNN methods, the prediction performance of our methods are improved significantly for detecting the low-quality records (“Too Noisy To Classify”). This is partly due to the improvement of class skewness (as the skewness-driven dynamic augmentation makes the proportion of records with different labels closer to each other, that is, the training records of “Too Noisy To Classify” increase greatly), and the improvement of noisy segments problem (as our method would be more attentive to the most important segments and avoid learning features from noise segments for all records, which could reduce, to some extent, the misclassification of “Too Noisy To Classify” records).

**Figure 7:**
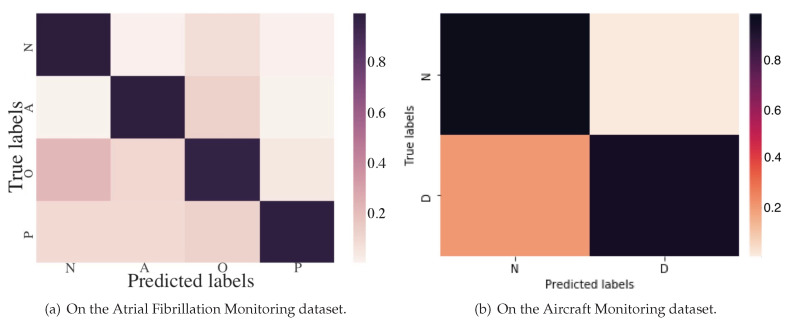
Confusion matrix of KEEN.

Moreover, the confusion matrix of the model predictions on the test dataset of Atrial Fibrillation Monitoring dataset is shown in Figure 7a. Mistakes made by models are almost understandable. To give just a few examples, many “Too Noisy To Classify” records are confused with “Other Rhythm” records which makes sense given that it can be subtle to detect “Other Rhythm” records, especially when their ECG morphologies are similar or when noise is present. In addition, it makes sense to confuse “Other Rhythm” records and “Normal Sinus Rhythm” ones, as it is difficult to distinguish among them sometimes, even for cardiologists.

#### 4.1.2. Interpretation Results of Atrial Fibrillation Diagnosis

**Figure 8:**
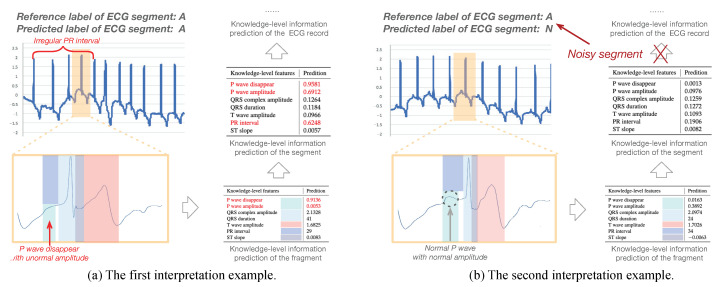
Case studies of prediction interpretation on the Atrial Fibrillation Monitoring dataset.

To show the interpretation results, we first evaluate the average error of all the knowledge-level attributes used in the experiments in Table 3. We can see that the average errors of all knowledge-level features are very small. Apart from quantitative analysis, we list two interpretation examples in Figure 8 to better understand how an interpretation model works. Each example contains one segment of the ECG record shown in Figure 1; the knowledge-level features generated by the KEEN+, thereafter, output them for the fragments, segments, and the ECG record (i.e., the final output of knowledge-level features are generated by aggregation operations to combine all the knowledge-level features of the segments which belong to the same ECG records into one unified feature vector).

In the first example, the reference label and the predicted label of the first input ECG segment are both “A” as shown in Figure 8a. Moreover, the knowledge-level information predicted on both fragment and segment reveal characteristics of Atrial Fibrillation (e.g., high probability of P wave disappear, high value of standard deviation coefficient on P wave amplitude, and PR interval which reflect irregular changes of them between different fragments of an ECG segment, etc.). Consequently, the KEEN+ can automatically predict this important domain knowledge-level information, which could be provided as the domain technological decision-making basis, for the whole ECG record without the help of domain experts. Moreover, the KEEN+ could help cardiologists to accurately locate the ECG fragments when cardiac arrhythmia happens by analyzing the abnormal knowledge-level information (e.g., P wave disappear) of each fragment.

As another example, the reference label and the predicted label of the second input ECG segment are “A” and “N”, respectively. Some knowledge-level information (e.g., normal P wave with normal amplitude), which agree with the clinical symptoms of Normal Sinus Rhythm other than Atrial Fibrillation and indicate that the second input ECG segment is a noisy segment, can be obtained on the fragments and segment of the input ECG segment as shown in Figure 8b. As a matter of fact, these noisy segments should be avoided when using aggregation operations to generate the knowledge-level information for an ECG record, which is exactly the goal of this work. This problem is well addressed by the KEEN+ model, since aggregation operation would be only applied on the selected top-K most confident segments for the given ECG record, and therefore output a more consistent and accurate knowledge-level information.

### 4.2. Effect of Application in the Field of Aircraft Structural Health

We evaluate the effect of application in the field of aircraft structural health on the Aircraft Monitoring dataset, which is collected by the piezoelectric sensor networks on real aircraft parts.

#### 4.2.1. Effectiveness of Aircraft Structural Damage Detection

The precision, recall, and $F_{1}$ results are shown in Figure 6b. We can see that the proposed KEEN and KEEN+ method with 0.8012 and 0.8005 $F_{1}$ scores outperform all of the most advanced deep learning methods for the task of aircraft structural damage detection by 2.99–10.71% and 2.99–10.62%, respectively.

Figure 7b shows the confusion matrix of the model predictions on the test dataset of the Aircraft Monitoring dataset. Obviously, quite a few records of “Structural Damage” are confused with “Normal” ones, which is mainly because some invisible cracks to the human eye are really very difficult to distinguish from the normal ones in the aircraft structural health-condition monitoring records.

#### 4.2.2. Interpretation Results of Aircraft Structural Damage Detection

**Figure 9:**
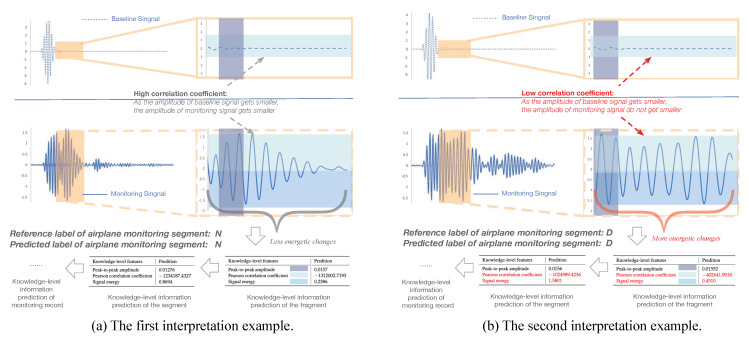
Case studies of prediction interpretation on the Aircraft Monitoring dataset.

The results of average error rates of the knowledge-level attributes used in the experiments are shown in Table 4, and we can see that the average errors of all knowledge-level features are very small as well. Similarly, we list two interpretation examples in Figure 9 to better understand how an interpretation model works in the task of aircraft structural damage detection. Each example contains one segment of an aircraft monitoring record, and each segment has two input channels—the baseline signal channel $x_{b}$ and the monitoring signal channel $x_{m}$.

The reference label and predicted label of the input aircraft monitoring segment shown in the first example are both “N”, while the reference label and predicted label are both “D” in the second example. Compared with the second example as shown in Figure 9b, the amplitude of monitoring signal in the first example (as shown in Figure 9a) decreases significantly, which is much more similar to the baseline signal, while the second one shows an even stronger amplitude variation which reflects higher signal energetic changes. The results indicate that the second example has a much lower Pearson correlation coefficient (measuring the correlation of amplitude variations between baseline signal and the monitoring signal) and higher signal energy (measuring the changes of energy), which meets the diagnostic criteria in aircraft structural damage.

### 4.3. Analysis of the Influence of Hyper-Parameters

To evaluate the effect of different hyper-parameter settings on the performance of our method, four main hyper-parameters (threshold *K*, window size W, maximum stride threshold M, and length of each fragment N) are further evaluated.

**Figure 10:**
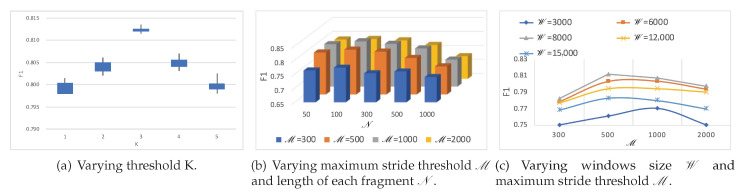
$F_{1}$ score results of KEEN for for different hyper-parameters.

Figure 10a demonstrates the effects of threshold K. The $F_{1}$ score increase when the threshold K varies within the range of [1,3], yet they drop when the threshold K is larger than 3. The primary cause is that the accuracy of the proposed model depends on both the number of segments engaged in the algorithm and the confidence of their labels. The threshold K not only determines how many segments of each record participate in the algorithm but also the confidence of each segment involved in the training process. That is, the higher the reasonable K value, the lower the confidence of predictions (as we choose K segments from the most confident one to the less confident one). However, a much higher K value may lead to a decrease in the confidence of segments participating in the learning and voting phase, and degrade the performance of the proposed model as well.

Furthermore, the effects of two main hyper-parameters (maximum stride threshold M and length of each fragment N) are shown in the Figure 10b. When parameters of W and N vary within the range of [500,1000] and [300,2000] respectively, smaller fragments N may benefit the overall performance. This is probably because it could provide more fragments for a recurrent neural network layer to capture trend-level features. However, more critical local morphological-level features might be lost in a ‘too small’ fragment.

In addition, when the hyper-parameters of windows size W and maximum stride threshold M change within the range of [3000,8000] and [300,2000], respectively, as shown on the Figure 10c, usually the smaller the parameters, the larger the augmented training data, and the better the performance of the proposed model. Nevertheless, if a ‘too small’ parameter of windows size W which could only provide a very narrow view for extracting local morphological-level features, the model performance might be degraded too.

### 4.4. Discussion

The K-margin-based intErpretable lEarNing (KEEN) is a general framework for health-condition assessment, which aims to provide interpretable health-condition detection results on a dataset with class skewness and noisy segments. The advantages of the proposed method are fourfold:

- It can automatically adapt to the dataset with class skewness (e.g., the Atrial Fibrillation Monitoring dataset) and improve the model performance on the dataset without class skewness as well by improving the inadequacy of well-labeled data (e.g., the Aircraft Monitoring Dataset) using the skewness-aware RCR-Net model.
- No extra process of signal noise elimination is needed to help the prediction of health conditions from monitoring data that contains noisy segments (both segments with too many signal noises and segments with noisy labels).
- Low-quality noisy records can be reliably detected, as our method would not cause the problem of changes or losses of features representing the quality of the monitoring signal itself.
- Important domain knowledge features can be extracted without the help of human experts from the time-series data automatically. As a result, KEEN+ can provide an interpretable decision-making basis or technological proof by using these domain knowledge-level features.

However, it has two disadvantages:

- It needs extra computation when filtering the noisy segments. Nevertheless, the computational cost, which is $\lceil \frac{W}{N}\rceil \times K$, is very low compared with the computational cost of DNN methods. For example, for the experimental settings mentioned in Section 3, the extra computational cost of each monitoring record is $\lceil \frac{6000}{300}\rceil \times 3$ = 60.
- The model performance could be influenced by the inaccuracy of the knowledge-level characteristics extracted from the dataset with high noise contamination. Comparing with the similar $F_{1}$ scores of KENN and KEEN+ on the Aircraft Monitoring Dataset, the accuracy of the KEEN+ method is slightly lower than that of the KEEN method on the Atrial Fibrillation Monitoring Dataset. This may be mainly because the noise contamination of the Atrial Fibrillation Monitoring Dataset is much higher, and thus the accuracy of the extracted knowledge-level characteristics is lower. From a performance perspective, the knowledge-level interpretation method does lower the model performance to some extent on the dataset with high noise contamination, but, considering that it can make results more explicable, this performance degradation is acceptable.

## 5. Conclusions

In this paper, we propose a KEEN model for health-condition assessment which can automatically handle the problems of class skewness and noisy segments, and provide knowledge-level interpretations of the predicted results that can be used as a decision-making basis as well. Through experimental validation in the field of both medical and aerospace, the proposed method has a better generality and effectiveness comparing with the state-of-the-art deep learning methods for health-condition assessment tasks. Moreover, the following conclusions can be drawn from the test scene:

- The proposed methods KEEN and KEEN+ with 0.8125 and 0.7974 $F_{1}$ scores, which outperform all state-of-the-art DNN methods for health-condition assessment tasks by 3.30% and 5.71% on the Atrial Fibrillation Monitoring dataset with class skewness and noisy segments, indicate that the proposed methods can automatically adapt to the dataset with class skewness and noisy segments.
- The KEEN (with 0.8012 $F_{1}$ scores) and KEEN+ (with 0.8005 $F_{1}$ scores) are able to perform 2.99% better by improving the inadequacy of well-labeled data and filtering the noisy segments on the Aircraft Monitoring Dataset without class skewness.
- KEEN and KEEN+ show good performance with 0.7561 and 0.6995 $F_{1}$ scores for detecting the low-quality records (“Too Noisy To Classify”) which outperform other DNN methods by 28.74% and 19.10% on the Atrial Fibrillation Monitoring dataset.
- The model interpretation shows improvement in the results compared to other DNN methods, as KEEN+ can provide domain knowledge-level features as technological proofs for the results of health-condition assessment.

In future work, it will be important to investigate how the proposed approach could be performed online (e.g., taking the skewness drift problem into account, how to rapidly update the model with a new data stream, etc.). Moreover, another possible rewarding avenue of future research is to consider multi-modality data input and more fine-grained output categories to improve the model performance and apply it in a more practical situation.

## Figures and Tables

**Table 1:** Counting rules for the numbers of the variables used in the $F_{1}$ evaluation measurements for Structural Damage Monitoring Dataset.

	Predicted Class			
**Reference Class**		Normal(N)	Structural Damage(D)	Total
	Normal(N)	$N_{n}$	$N_{d}$	∑N
	Structural Damage(D)	$D_{n}$	$D_{d}$	∑D
	Total	∑n	∑d	
	……			

**Table 2:** Comparing our method with other deep learning methods on the Atrial Fibrillation Monitoring dataset.

Method	$F_{1N}$	$F_{1A}$	$F_{1O}$	$F_{1P}$	$F_{1}$
CNN	0.9123	0.8140	0.7097	0.4636	0.7249
RNN	0.9187	0.8100	0.7834	0.4829	0.7488
RCNN	0.9190	0.8221	0.7319	0.5676	0.7602
ResNet	0.9056	0.8431	0.7415	0.5873	0.7719
KEEN	0.9061	0.8712	0.7166	0.7561	0.8125
KEEN+	0.9053	0.8704	0.7142	0.6995	0.7974

**Table 3:** Error analysis of knowledge-level features on the Atrial Fibrillation Monitoring dataset.

Knowledge-Level Features	Average Error
P wave disappear	0.3918
P wave amplitude	0.0540
QRS complex amplitude	0.009
QRS duration	0.021
T wave amplitude	0.042
PR interval	0.196
ST slope	0.017

**Table 4:** Error analysis of knowledge-level features on the Aircraft Monitoring dataset.

Knowledge-Level Features	Average Error
Peak-to-peak amplitude	0.0124
Pearson correlation coefficient	0.1397
Signal energy	0.0024

## Abbreviations

The following abbreviations are used in this manuscript:

DNN	Deep Neural Network
CNN	Convolutional Neural Network
RNN	Recurrent Neural Network
CRNN	Convolutional Recurrent Neural Network
ResNet	Residual Neural Networks
Bi-LSTM	Bi-Directional Long-Short Term Memory
RCR-Net	Residual-Convolution-Recurrent Neural Network
K-margin	K-Margin-Based Health-Condition Label Prediction Algorithm
KEEN	K-Margin-Based Interpretable Learning
MSE	Mean Square Error Loss
ECG	Electrocardiograph
AF	Atrial Fibrillation
